# Temporary CBD Stenting with a Nelaton Tube Is a More Practical and Safer Option Than T-Tube Drainage after Conventional CBD Exploration for Choledocholithiasis

**DOI:** 10.1155/2018/8035164

**Published:** 2018-09-13

**Authors:** Ashraf M. Abdelkader, Ahmed M. Zidan, Mohamed T. Younis

**Affiliations:** General Surgery Department, Faculty of Medicine, Banha University, Egypt

## Abstract

**Objective:**

We are trying to investigate the possibility, safety, and benefits of replacing the role of T-tube by another more safe and effective procedure for biliary decompression in the case of common bile duct (CBD) exploration.

**Methods:**

Our present study includes fifty consecutive patients who underwent a traditional CBD exploration due to choledocholithiasis. Patients were divided into 2 equal groups. In the 1^st^ group, a spontaneously expelled Nelaton tube is placed in the CBD to aid in bile drainage to the duodenum, while in the 2^nd^ group, a conventional T-tube is placed to decompress the CBD in the early postoperative (PO) days to a drainage bag. Operative and PO data as well as PO hospital stay time were recorded. All data were collected and statistically analyzed.

**Results:**

The mean operative time and PO hospital stay days were significantly low (p value < 0.05) in the Nelaton tube drainage group compared with the T-tube drainage group. On the other hand, the mean time needed for the abdominal drain removal was significantly higher in the T-tube drainage group (p value < 0.05).

**Conclusion:**

Nelaton tube with internal biliary drainage is effective and safer than T-tube drainage and it helps in reduction of the PO hospital stay time. In addition, it avoids all short-term complications of T-tube.

## 1. Introduction

Choledocholithiasis is the 2^nd^ most common complication of gallbladder stone disease and its frequency is about 10–20 % in symptomatic gallstones as well as in 5 % of asymptomatic patients [1, 2]. Extraction of CBD stones is mandatory to avoid additional complications such as obstructive jaundice, pancreatitis, and cholangitis [3]. The ideal line of dealing with choledocholithiasis is quite unclear, and the feasible choices are open CBD exploration, laparoscopic CBD exploration, and endoscopic retrograde cholangiopancreatography (ERCP) combined with or followed by laparoscopic cholecystectomy (LC) [4]. Placement of a T-tube for biliary decompression is a well-established surgical method done for controlling bile flow and pressure in the CBD and minimizing the postoperative (PO) bile leakage through the suture line until the swelling and oedema at the ampulla of Vater to subside after CBD exploration [5]. Moreover, T-tube drainage allows an easy PO cholangiography and removal of residual CBD stones. [6].

Routine placement of T-tube for bile drainage is still a point of controversy among surgeons. T-tube placement is defined to diminish PO pressure and oedema in CBD and may encourage the healing process [3, 7]. However, many reasons mentioned against the routine use of T-tube in the form of longer hospital stay, physical discomfort, the delayed return to ordinary activity and work, and the risk of track infection increase the need for analgesics, displacement of the tube, cholangitis, and bile leakage for a long time after T-tube removal. All these reasons in addition to other long-term complications could lead to high PO morbidity and mortality [2, 8]. However, in the developing countries with the deficiency of the economic resources where ERCP is not available to a high extent, the need for CBD exploration for stone extraction is still indicated in many regions. Due to this argument about the hazards of T-tube, we offered in this study a cheaper alternative and brilliant method that avoids insertion of a T-tube after conventional CBD exploration.

## 2. Materials and Methods

This study was completed at the General Surgery Department, at Benha University Hospital in Egypt, and King Saud Hospital in Saudi Arabia from March 2016 until March 2018. The study includes 50 consecutive patients who are a candidate for CBD exploration due to CBD stones, after approval of the study procedure by the Ethical Committee. Patients were informed in detail of the possible risks and advantages of the 2 procedures (placement of a segment of a Nelaton catheter as a stent in CBD or T-tube drainage). Also, fully informed written consent for surgical operation and the participation in the study was obtained.

Patients were admitted through the outpatient department (OPD) or emergency room (ER) and then assisted through clinical, laboratory, and radiological evaluation. Patients were examined generally beside local abdominal examination. Laboratory tests by means of CBC, blood sugar, liver enzymes, serum bilirubin (conjugated, unconjugated, and total), alkaline phosphatase (ALP), Gamma-Glutamyl Transferase (GGT), and kidney function tests were done. Diagnostic imaging was done in the form of abdominal ultrasonography (US), computed tomography (CT) scans, and magnetic resonance cholangiopancreatography (MRCP) to confirm the presence of CBD stones. In most cases of choledocholithiasis in our study, preoperative ERCP was not possible as we do not have ERCP in our hospital and patients cannot be transferred to other institutes due to financial issues. Only 5 cases underwent preoperative ERCP; however, ERCP failed to remove large and/or multiple CBD stones. Inclusion criteria for this study include patients who are diagnosed as having CBD stones with absence of ERCP facility or failure of stone extraction through ERCP, age >18 years and ≤ 70 years, ability to sign a consent form and undergo the study procedure, and American Society of Anesthesiology (ASA) score of I-III. Exclusion criteria include patient participation in another study, ASA score > III, and prominent psychiatric disease.

Patients were divided into 2 groups (25 patients in each group) according to the type of biliary drainage procedure as either Nelaton tube drainage group or T-tube drainage group. In this study, we used the “alternation” method as an allocation process that is not subject to anyone's personal decision. In this distribution method, we did Nelaton tube drainage to the 1^st^ patient who was included in the study, then T-tube drainage to the 2^nd^ patient, then Nelaton tube drainage to the 3^rd^ patient, and so on. The collected data included (1) preoperative demographic data including gender, age, ASA score, the diameter of the CBD, and associated coexisting diseases; (2) operative and PO data including operative time and intraoperative blood loss, time needed to regain bowel movements, PO bile leak, time to remove the abdominal drain, and the length of PO hospital stay days. The 1'ry endpoint included assessment of the operative time, PO bile leak, and the length of hospital stay. Data was collected at each participating hospital, then compared, and analyzed.

*Preoperative*. Thromboembolic prophylactic measures were done and patients received a single dose of prophylactic IV antibiotic (Metronidazole 500 mg and Ceftriaxone 1 g) one hour before surgery. 

*Operative Technique*. All operations were done under general anesthesia. Surgery was completed by a right subcostal incision. Dissection and clearance were done to the anterior wall of CBD, cystic duct, and common hepatic duct (CHD). Longitudinal choledochotomy (1-2 cm) was done in the midline of the anterior wall of CBD just distal to cystic duct opening. Extraction of CBD stone/s was done with the help of stone forceps. Irrigation of CBD, CHD, and the two hepatic ducts was with normal saline through 50 ml syringe and a tube of appropriate size. Smooth dilatation of distal CBD and sphincter of oddi was done with a dilatorVater through pushing saline to see and palpate duodenal saline to see and palpate duodenal distension with saline.

**Figure 1:**
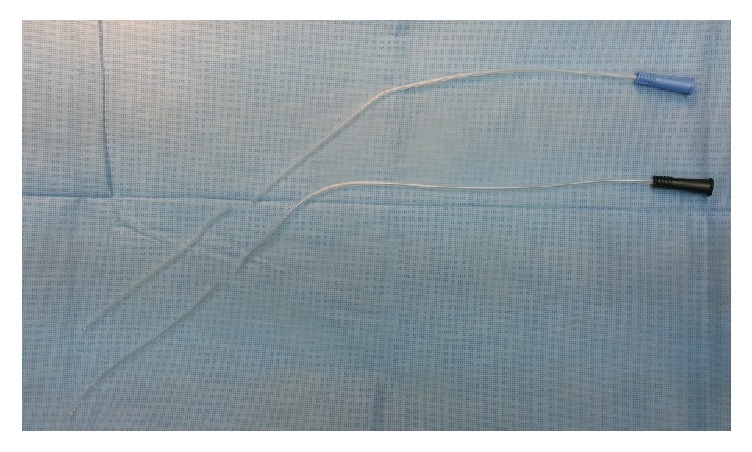
Nelaton tubes of 8 and 10 French size.

*In the Nelaton tube drainage group*, the distal segment (8-10 cm) of Nelatone tube of 8 or 10 French size with blue or black base, respectively, (Figure 1) was cut and fashioned with multiple side openings along its length and placed inside CBD to be extended 2 cm above the choledochotomy level as well as up to 2-3 cm inside the duodenum. The choledochotomy is closed in 3-4 sutures with Vicryl 4/0 over a rounded needle. The Nelatone tube was included in one suture to be fixed in place for an appropriate time (the time needed for complete resorption of Vicryl: 30-60 days). Then, cholecystectomy was done and drain kept in the Morison's pouch.

*In the T-tube drainage group*, after clearance of the CBD and cholecystectomy was completed, another small (5 mm) opening was made in the CBD or CHD proximal to the 1^st^ cholodoctomy for T-tube insertion. A T-tube of appropriate size was fashioned and inserted in its place and the other end was passed through the abdominal wall and fixed to the skin. A drain kept in Morison's pouch and abdominal wall closed in layers. 

*Postoperative Care*. The antibiotic was sustained for one day after surgery. Mechanical and chemical thromboembolic prophylactic measures were maintained according to protocols. Soft fat-free diet was allowed on the 1^st^ PO day. 

*The Nelaton Tube Drainage Group*. The abdominal drain was removed between the 2^nd^ and 3^rd^ PO days when the drainage became minimal serous fluid (< 30 ml in 24 hours). Patients were discharged home between the 3^rd^ and 5^th^PO day. The Nelaton tube stent was spontaneously expelled with the stool in 23/25 patients within 60 days after surgery as observed by patients themselves. For the remaining 2/25 patients, an abdominal X-ray+US was done after 60 days to confirm the site of the tube. If a part of the Nelaton tube is still inside the CBD, the patient is prepared for upper gastrointestinal endoscopy for tube extraction by grasping its distal end (which was visualized inside the duodenum). 

*The T-Tube Drainage Group*. Abdominal drain was removed between the 2^nd^ and 5^th^PO days once the drainage became minimal serous fluid (< 30 ml in 24 hours). Through the T-tube, a cholangiography was done on the 14^th^ PO day to exclude any stricture or residual CBD stones. The T-tube was removed after confirmation of normal cholangiography. Frequent dressing on the skin opening at the site of T-tube is till stopping of discharge. Patients were discharged home between the 15^th^ and 16^th^ PO day. Follow-up was done in the OPD after discharge till patients was completely improved.

### 2.1. Statistical Analysis

The data presented as mean ± SD, numbers, ranges, and ratios. The results were analyzed by means of Wilcoxon's ranked test. Statistical analysis was implemented using the SPSS version 21 (IBM Corp., Armonk, NY, USA) for Windows statistical package. The P value was considered statistically significant if < 0.05.

## 3. Results

The study contained 50 patients with CBD stones whose underwent a conventional CBD exploration. Patients were divided into 2 equal groups (25 patients in each group) according to the procedure of biliary drainage, the Nelaton tube drainage group, and the T-tube drainage group. The was no difference regarding gender, age, and ASA score among patients of both groups. Clinical examination findings, medical history, and preoperative diameter of CBD also were not statistically different among both groups (Table 1).

All patients in the two groups passed the operations without any major operative complications. The operative time in the Nelaton tube drainage group was significantly lower (p value < 0.05) than T-tube drainage group. The mean time needed for the abdominal drain removal was significantly higher in the T-tube drainage group (p value < 0.05). The mean postoperative hospital stay was significantly lower (p value < 0.05) among patients of the Nelaton tube drainage group (Table 2).

## 4. Discussion

Agreement on the ideal method for the treatment of CBD stones is still a point of controversy till now. If possible, preoperative ERCP and sphincterotomy followed by laparoscopic cholecystectomy are the most popular option for the management of this disease nowadays [9]. Nevertheless, ERCP and sphincterotomy are associated with various biliary complications in 8–10 % of patients [10]. Inaccessibility of ERCP is due to financial issues and incapability of laparoscopic CBD exploration is due to lack of facilities in several regions of our developing countries; all these factors make us forced to deal with choledocholithiasis with traditional methods through an open CBD exploration. On the other hand, exploration of the CBD was associated with an obligatory insertion of a decompressing biliary drain. However, a frequent complication of T-tube insertion which was mentioned before it has been documented with a frequency of about 10–15 % [11]. On the other hand, primary CBD closure without a biliary drainage procedure after CBD exploration for choledocholithiasis has been performed by several institutes for many years [5, 6]. This may avoid the drawbacks of T-tube placement; however, it carries a high risk of biliary leak with the need for other interventions to solve this serious problem [12]. Muzaffar et al., in their study that compare the 1ry CBD clouser with the T-tube insertion, found that the complications among patients of the 1ry closure were lesser than that in the T-tube group. Moreover, they mentioned that, during open CBD surgery for stones, 1ry closure of the CBD seemed to be effective and safe with shorter postoperative hospital stays [13].

Our technique (Nelatone tube drainage) tried to avoid all complications of T-tube drainage and at the same time, and it keeps all advantages of biliary drainage after CBD exploration. This procedure proved that the use of a cheap and frequently available tube as a spontaneously expelled CBD stent drainage is fairly economical and more simple with lesser intraoperative blood loss and shorter operative time and offers a significantly shorter PO hospital stay time. There is no doubt that using T-tube drainage after CBD exploration will lead to loss of bile and loss of electrolytes, disturb the absorption function of the bowel, and slow down the intestinal peristalsis [12]. This was clearly manifested in our study by a significant delay in bowel movements and delayed passage of flatus or faces in patients of the T-tube drainage group. On the contrary, with the Nelaton tube biliary stent, the biliary pressure decompressed without loss of bile, and this may explain the early PO regain of bowel movements and decrease in the PO morbidities [14]. Fortunately, in our study, among patients with Nelaton tube biliary stent group, none of our patients manifested any biliary leakage.

In our opinion, one of the most important advantages of Nelaton tube biliary stent is the exit of this tube automatically with the stool between the 30 and the 60 PO days in 23/25 (92%) of cases without the need for any further interventions from the doctor or the need for the presence of the patients within the hospital; this leads to a reduction in the number of hospital stay days and reduction in total costs of the treatment. In the remaining 2/25 (8%) of the Nelaton tube group, the tube did not exit spontaneously within 60 PO days (the time expected for complete resorption of the fixing Vicryle suture) and removal of the tube was completed through an upper GIT endoscopy as a one-day procedure. On the other hand, in our study, the average time needed for removal of the T-tube was between 14 and 16 days, and during this time patients were still admitted in the hospital according to protocols that do not allow patients to go home with any abdominal drainage tube.

It is well known to us that, in order to avoid PO bile leakage at the suture line of cholodocotomy site, biliary decompression should be taken into consideration as a last step in CBD exploration surgery due to the expected temporary edema and obstruction at the distal end of the CBD in the 1^st^ few PO days (10-15 days) as a result of manipulations to extract the retained calculi as well as repeated attempts for dilatation of the distal CBD [15, 16]. We consider that the time needed for the spontaneous exit of Nelaton tube depends on the PO patient's physical activity, food habit, and gut motility. Moreover, other studies have reported that, in patients with bowel diverticulum or adhesions, intestinal perforation is more likely to happen as a result of stent migration regardless of the length, size, or type of the stent [17]. Therefore, use of Nelaton tube stent drainage is better to be avoided in patients with intestinal diverticulum or adhesions.

The spontaneously expelled Nelaton tube has succeeded in overcoming the hazards of the T-tube biliary drainage after open CBD exploration for patients with choledocholithiasis. This success may direct us to use a Nelaton tube as a biliary drainage method in case of iatrogenic or traumatic CBD injuries that need primary suturing; however, this step mandates a separate prospective study. Despite the marvellous outcomes and the statistical significance of our study results, more studies are mandatory to offer strong data about this procedure.

## 5. Conclusion

Using a Nelaton catheter tube as an internal biliary drainage after open CBD exploration in patients suffering choledocholithiasis is more safe and practical than T-tube external drainage method. Of course, an additional work is mandatory to study the possibility of using the Nelaton tube after primary suturing of CBD in case of laparoscopic CBD exploration as well as the iatrogenic and traumatic CBD injuries.

## Figures and Tables

**Table 1:** Patients' demographic data.

**Patients' data**	**Strata**	**Nelaton tube group**	**T-tube group**	**P-value**
**N (**%**)**		25(50%)	25(50%)	

**Age (years)**		41.21±8.12(23-67)	40.62±7.51(19-65)	0.762

**Sex**	Male	11(44%)	10(40%)	NS
	Female	14(46%)	15(60%)	NS

**ASA score**		1.58±0.61(1-3)	1.61±0.50(1-3)	0.623

**Diameter of CBD**		9.95+1.6(8-16.5)	10.2+1.73(9-17.4)	0.752

**Clinical signs**	Fever	4(16%)	3(12%)	NS
	Jaundice	17(68%)	18(72%)	NS

**Chronic co-exiting disease** **∗**	Hypertension	5(20%)	6(24%)	NS
	Diabetes	5(20%)	4(16%)	NS
	IHD	1(4%)	1(4%)	NS
	COPD	2(8%)	1(4%)	NS
	CLD	1(0%)	0(0%)	NS

Data are presented as mean ± SD & numbers; ranges and percentages are in parenthesis. ASA: American Society of Anesthesiologists; IHD: Ischemic heart disease; COPD: chronic obstructive pulmonary disease; CLD: chronic liver disease; CBD: Common bile duct; NS: nonsignificant. *∗*Some cases had more than one coexisting disease.

**Table 2:** Operative and 30-day postoperative data.

**Data**	**Nelaton tube group**	**T-tube group**	**P-value**
	**N=25**	**N=25**	
**Operative time (min)**	65±20.5 (80-130)	90±17.32 (95-170)	< 0.05

**Intra-operative blood loss (ml)**	73±25.63 (50-120)	75±32.85 (50-150)	NS

**Time for abdominal drain removal (days)**	1.6+0.8 (2-3)	2.3 ± 2.4 (2–5)	< 0.05

**Time to regain bowel motion**	1.2±0.52 (1-3)	2.4±1.35 (2-4)	< 0.05

**Bile leakage n (**%**)**	0	1 (4%)	NS

**PO hospital stay (days)**	2.5 +1.7 (3-5)	12.5 +3.62 (15-17)	< 0.05

Data are presented as mean ± SD & numbers; ranges and percentages are in parenthesis. PO: postoperative.

## Data Availability

The data used to support the findings of this study are included in the article or from the corresponding author upon request.

## References

[1] Shively E. H., Richardson M., Romines R., Englund G., Watkins J. (2010). Laparoscopic common duct exploration in 90-bed rural hospital. *The American Surgeon*.

[2] Savita K. S., Bhartia V. K. (2010). Laparoscopic CBD Exploration. *Indian Journal of Surgery*.

[3] Ahmed I., Pradhan C., Beckingham I. J., Brooks A. J., Rowlands B. J., Lobo D. N. (2008). Is a T-tube necessary after common bile duct exploration?. *World Journal of Surgery*.

[4] Abellán Morcillo I., Qurashi K., Abrisqueta Carrión J., Martinez Isla A. (2014). Laparoscopic Common Bile Duct Exploration. Lessons Learned After 200 Cases. *Cirugía Española (English Edition)*.

[5] Gurusamy K. S., Koti R., Davidson B. R. (2013). T-tube drainage versus primary closure after laparoscopic common bile duct exploration.. *Cochrane Database of Systematic Reviews*.

[6] Williams J. A., Treacy P. J., Sidey P., Worthley C. S., Townsend N. C., Russell E. A. (1994). Primary duct closure versus t-tube drainage following exploration of the common bile duct. *ANZ Journal of Surgery*.

[7] Ambreen M., Shaikh A. R., Jamal A., Qureshi J. N., Dalwani A. G., Memon M. M. (2009). Primary closure versus T-tube drainage after open choledochotomy. *Asian Journal of Surgery*.

[8] Zhang W., Li G., Chen Y. (2017). Should T-Tube Drainage be Performed for Choledocholithiasis after Laparoscopic Common Bile Duct Exploration? A Systematic Review and Meta-Analysis of Randomized Controlled Trials. *Surgical Laparoscopy, Endoscopy & Percutaneous Techniques*.

[9] Zhang W., Xu G., Huang Q. (2015). Treatment of gallbladder stone with common bile duct stones in the laparoscopic era. *BMC Surgery*.

[10] Iida T., Kaneto H., Wagatsuma K. (2018). Efficacy and safety of endoscopic procedures for common bile duct stones in patients aged 85 years or older: A retrospective study. *PLoS ONE*.

[11] Ibrahim T., Murat K. (2017). Retrospective clinical study of the effects of T-tube placement for bile duct stricture. *Medical Science Monitor*.

[12] Podda M., Polignano F. M., Luhmann A., Wilson M. S. J., Kulli C., Tait I. S. (2016). Systematic review with meta-analysis of studies comparing primary duct closure and T-tube drainage after laparoscopic common bile duct exploration for choledocholithiasis. *Surgical Endoscopy*.

[13] Hashmi A. M., Khawaja I. S., Butt Z. (2014). The Pittsburgh sleep quality index: validation of the Urdu translation. *Journal of the College of Physicians and Surgeons Pakistan*.

[14] Lee J. S., Yoon Y. C. (2016). Laparoscopic common bile duct exploration using V-Loc suture with insertion of endobiliary stent. *Surgical Endoscopy*.

[15] Huang J., Zhu J. (2009). Spontaneously removed endobiliary J stent drainage after laparoscopic common bile duct exploration. *Surgical Endoscopy*.

[16] Xu Y., Dong C., Ma K. (2016). Spontaneously removed biliary stent drainage versus T-tube drainage after laparoscopic common bile duct exploration. *Medicine (United States)*.

[17] Lenzo N. P., Garas G. (1998). Billiary stent migration with colonic diverticular perforation. *Gastrointestinal Endoscopy*.

